# Effects of Epstein-Barr Virus Infection on CD19+ B Lymphocytes in Patients with Immunorelated Pancytopenia

**DOI:** 10.1155/2020/4098235

**Published:** 2020-02-17

**Authors:** Yang Zhao, Yihao Wang, Hui Liu, Kai Ding, Chunyan Liu, Hong Yu, Zonghong Shao, Rong Fu

**Affiliations:** Department of Hematology, Tianjin Medical University General Hospital, China

## Abstract

**Objectives:**

To explore effects of Epstein-Barr virus (EBV) infection on CD19+ B lymphocytes in patients with immunorelated pancytopenia (IRP).

**Methods:**

An enzyme-linked immunosorbent assay (ELISA) in vitro diagnostic kit was used to detect EBV capsid antigen- (CA-) IgG and VCA-IgM antibodies in the serum. We analyzed the EBV-DNA copies of CD19+ B lymphocyte by using real-time quantitative polymerase chain reaction (RT-qPCR). CD21, CD23, CD5, CD80, and CD86 receptors on the surfaces of CD19+ B cells were detected by flow cytometry (FCM). The correlation between these receptors and EBV-DNA copies were evaluated.

**Results:**

The results revealed that the positive rate of EBVCA-IgM and CD19+ B lymphocyte EBV-DNA copy in the IRP group were significantly higher than those in the control group (*P* < 0.05). CD19+ B lymphocyte EBV-DNA copies were also more abundant in IRP patients than in control subjects (*P* < 0.05). CD19+ B lymphocyte EBV-DNA copies were also more abundant in IRP patients than in control subjects (*P* < 0.05). CD19+ B lymphocyte EBV-DNA copies were also more abundant in IRP patients than in control subjects (

**Conclusions:**

EBV infection may activate CD19+ B lymphocytes and further disrupt bone marrow hematopoiesis in IRP patients.

## 1. Introduction

Immunorelated pancytopenia (IRP) is a type of hemocytopenia regarded as an autoimmune disease that is caused by unknown autoantibodies, which may suppress hematopoietic cells in the bone marrow, leading to anemia, bleeding, and infection [1]. IRP exhibits the following features: (i) hemocytopenia with a normal or higher than normal percentage of reticulocytes and/or neutrophils; (ii) hyperplasia in the bone marrow, exemplified by a higher percentage of nucleated erythroid cells in the sternum, with erythroblastic islands that are easily observed; (iii) good patient response to corticosteroids or high-dose intravenous immunoglobulin; (iv) exclusion of other primary and secondary hemocytopenia disorders; and (v) positive result in the BMMNC-Coombs test (bone marrow mononuclear cell Coombs test) [2–4]. At present, IRP pathogenesis is considered to result from abnormalities in the number, subsets, function, and activation of B lymphocytes [5].

The Epstein-Barr virus (EBV) belongs to a class of viruses with double-stranded DNA that are hosted by B lymphocytes. These viruses can interfere with immune function and stimulate cell proliferation and transformation [6, 7]. EBV is thought to be an environmental trigger of, and one of the principal candidates that causes, autoimmune diseases. Accordingly, EBV is associated with autoimmune diseases such as systemic lupus erythematosus (SLE), rheumatoid arthritis (RA), multiple sclerosis (MS), autoimmune thyroiditis, inflammatory bowel disease, insulin-dependent diabetes mellitus, Sjögren's syndrome, systemic sclerosis, myasthenia gravis, and autoimmune liver disease [8].

However, whether EBV infection affects autoimmune responses via B lymphocytes in IRP patients remains unknown. In this study, we determined levels of EBV antibodies and EBV-DNA copy numbers in IRP patients and normal controls. The abundances of the CD21, CD23, CD5, CD80, and CD86 receptors on the surfaces of CD19+ B cells were analyzed to elucidate the role of EBV in IRP pathogenesis.

## 2. Materials and Methods

### 2.1. Patient Description

A total of 72 IRP patients (42 females and 30 males; median age, 39 years; age range, 16–72 years) were enrolled in this study. All subjects were inpatients at the Department of Hematology, Tianjin Medical University General Hospital (Tianjin, China), between January 2017 and June 2018 and diagnosed according to Fu et al. [2]. Patient responses were evaluated according to the criteria for aplastic anemia. Patients were considered in remission if they met the following criteria: (i) disappearance of anemia and hemorrhagic symptoms; (ii) hemoglobin levels reaching 120 and 100 g/L in males and females, respectively; (iii) white blood cell counts reaching 3.5 × 10^9^ cells/L; and (iv) increase in platelet count. The IRP patients were divided into two groups based on results of EBV capsid antigen- (CA-) IgM assays: (i) anti-EBVCA IgM negativity and (ii) anti-EBVCA IgM positivity. A total of 36 healthy volunteers (20 females and 16 males; median age, 40 years; age range, 20–68 years) with normal blood picture and immune parameters were selected as normal controls.

### 2.2. Serological Diagnosis of EBV Infection

An enzyme-linked immunosorbent assay (ELISA) *in vitro* diagnostic kit (Euroimmun Medical Diagnostics, Lübeck, Germany) was used to detect EBV VCA-IgM antibodies and EBV VCA-IgG antibodies in the serum [9, 10]. ELISA was performed according to the manufacturer's instructions. The absorbance was measured at a wavelength of 450 nm and a reference wavelength of 630 nm. The signal-to-cutoff ratio (S/CO) of specimens > 1.1 was considered as positive and <0.8 as negative and <0.8 but <1.1 was equivocal.

### 2.3. Purification of CD19+ B Lymphocytes Using MACS Microbead Technology

Peripheral blood mononuclear cells (PBMCs) were isolated from the venous blood of IRP patients and controls treated with ethylenediaminetetraacetic acid (EDTA) anticoagulated using Ficoll-Hypaque density gradient centrifugation. Blood samples were diluted at 1 : 1 in Lymphocyte Separation Medium (Solarbio Science & Technology, Beijing, China) and centrifuged at 2,200 rpm and 25°C for20 min. The material at the interface between layers was collected and washed with phosphate-buffered saline (PBS) at 1,500 rpm for 10 min; then the supernatant was completely aspirated. PBMCs were resuspended in batches of 10^7^ cells in 80 *μ*L buffer and 20 *μ*L CD19 MicroBeads (Miltenyi Biotech, Bergisch Gladbach, Germany) and then incubated in the refrigerator at 4°C for 15 min. Finally, cells were washed with 2 mL buffer and resuspended in up to 500 *μ*L buffer. The MS column was placed in the magnetic field of the MACS separator. After the column was prepared by rinsing with 1 mL buffer, the cells were added to the column. The column was washed with 1.5 mL buffer, and all flow-through containing unlabeled cells were collected. Magnetically labeled cells were flushed out by firmly pushing the plunger into the tube, and CD19+ B lymphocytes were harvested.

### 2.4. Real-Time Quantitative Polymerase Chain Reaction (RT-qPCR)

Total DNA was extracted from 1 × 10^6^ sorted CD19+ B cells, and a quantitative diagnostic kit for EBV DNA (Beijing SinoMDgene Technology Co., China) was used to acquire the DNA copy numbers of the EBV BamH1W gene via PCR-fluorescence probe reaction.

### 2.5. Flow Cytometry (FCM) Analysis

Fresh peripheral blood (100 *μ*L per tube) treated with ethylenediaminetetraacetic acid anticoagulant was washed three times with PBS. Samples were then divided into one control and seven treatment tubes. Cells were stained with antibodies against mouse IgG1-fluorescein isothiocyanate (FITC), mouse IgG1-phycoerythrin (PE), and mouse IgG1-allophycocyanin (APC; BD Biosciences, Franklin Lakes, NJ, USA) as a negative control. Treatment cells were stained with antibodies against CD19-APC, CD5-FITC, CD23-PE, CD80-PE, CD86-PE, *κ*-FITC, and *λ*-PE (BD Biosciences) in separate tubes. One treatment tube contained cells stained with antibodies against CD19-FITC and CD21-APC. After the tubes were incubated in the dark at 4°C for 30 min, 2 mL erythrocyte lytic solution was added to each tube, and the tubes were incubated again at room temperature for 10 min. Subsequently, the cells were washed twice with PBS. At least 10^4^–10^5^ cells were acquired from each tube and analyzed using a fluorescence-activated cell sorter (FACS) flow cytometer (Beckman; US).

### 2.6. ELISA

Serum levels of soluble CD23 (sCD23) in IRP and control subjects were measured using an ELISA reagent kit (CloudClone, USA) according to the manufacturer's protocol. Diluted standards and 100 *μ*L serum from each patient were added in duplicate to the assay plate, which was incubated at 37°C for 2 h. After washing the plate five times, 100 *μ*L of antibodies was added to each well and the plate was incubated again for 90 min; then horseradish peroxidase was added to each well. The plate was incubated at 37°C for 30 min, and each well was washed five times. Subsequently, tetramethylbenzidine solution was added to each well and the samples were incubated in the dark at room temperature for 20 min. Finally, a stop solution was added, and the optical density of each mixture at 450 nm was read within 15 min.

### 2.7. Statistical Analyses

The IRP patients were divided into two groups according to the results of EBV-antibody assays: (i) anti-EBVCA IgM negativity and (ii) anti-EBVCA IgM positivity. The white blood cell and platelet counts and hemoglobin levels of these patients were analyzed. Patient follow-up was conducted via telephone to check routine blood parameters 1 year after patients were discharged to calculate their remission rate. We also estimated the time it took for leukocyte, hemoglobin, and platelet levels to reach levels indicative of remission.

All statistical analyses were performed using SPSS software (ver. 22.0; IBM Corporation, Armonk, NY, USA). The data are presented as the mean ± standard deviation (SD) for normally distributed data, and comparisons between two independent samples were performed using the *t*-test. For skewed distributions, median and interquartile spacing were calculated and compared using the rank-sum test. Constituent ratios were compared using the chi-squared test, and data were correlated using Spearman's rank test. Differences were considered statistically significant at *P* < 0.05.

### 2.8. Patient and Public Involvement Statement

The current study was approved by the Ethical Committee of Tianjin Medical University, and written informed consent was issued by the patients for the publication of this study.

## 3. Results

### 3.1. EBV Infection Rates in IRP Patients

This study examined 72 patients with IRP and 36 control subjects without IRP. EBVCA-IgG was detected in 98.6% (71 of 72) of IRP patients. The detection rate of EBVCA-IgG in control subjects was 97.2% (35 of 36). Thus, no significant differences were observed between the two groups (*χ*^2^ = 0.254, *P* = 0.613). EBVCA-IgM was detected in 43.1% (31 of 72) of IRP patients and 22.2% (8 of 36) of control subjects. Thus, EBVCA-IgM-positive rate was significantly higher in IRP patients than in control subjects (*χ*^2^ = 4.515, *P* = 0.033; Table 1).

EBV infection status also differed between IRP and control subjects, based on the positive rate of EBV-DNA copies. IRP patients exhibited a higher rate of EBV lytic infection compared to control subjects, with 45.1% (23 of 51) of IRP patients harboring EBV-DNA copies compared to 22.2% (6 of 27) of control subjects (*χ*^2^ = 3.955, *P* = 0.047; Table 2).

**Figure 1:**
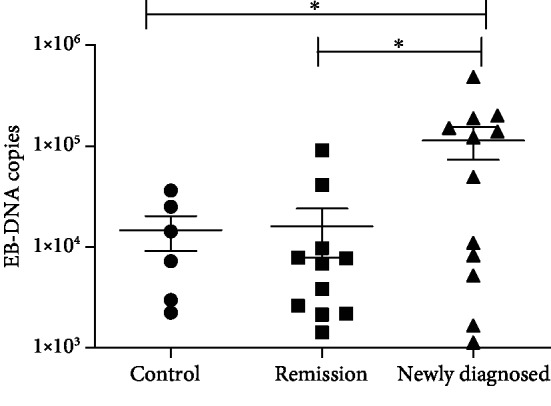
Numbers of CD19+ B lymphocyte EBV-DNA copies in patients with immunorelated pancytopenia (IRP) and control subjects. EBV: Epstein-Barr virus. ^∗^*P* < 0.05.

CD19+ B lymphocyte EBV-DNA copy numbers in newly diagnosed IRP patients (median, 9.646 × 10^3^; range, 2.660 × 10^3^–1.5475 × 10^5^) were significantly higher than those in patients in remission (median, 7.781 × 10^3^; range, 2.191 × 10^3^–4.1210 × 10^4^) and those in control subjects (median, 7.277 × 10^3^; range, 2.557 × 10^3^–1.9775 × 10^4^; Table 3; Figure 1).

### 3.2. Antigen Expression on B Lymphocyte Surfaces in EBV-Infected IRP Patients

**Figure 2:**
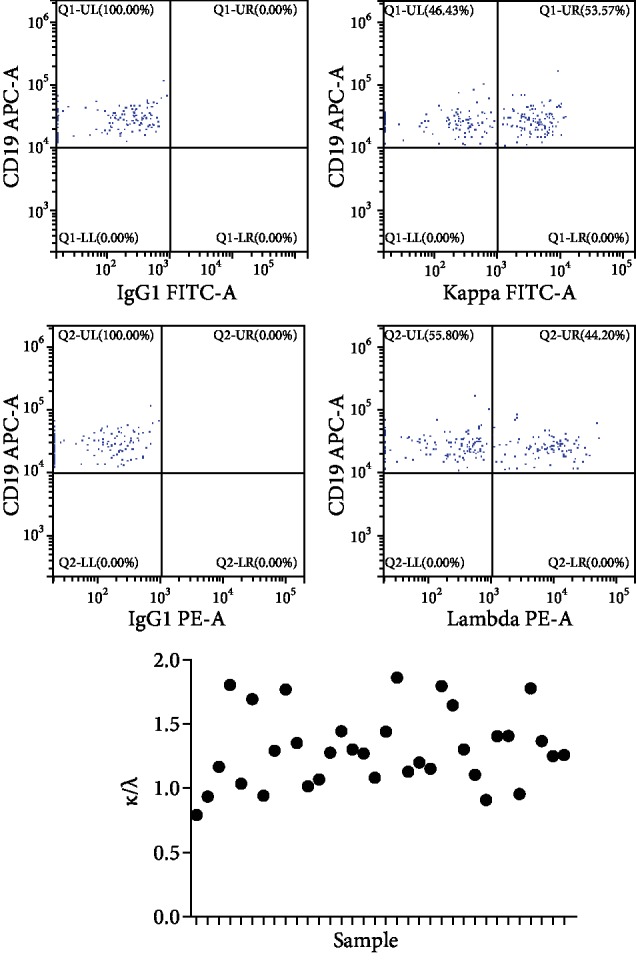
Kappa : lambda ratios (*κ*/*λ*) for B lymphocytes in IRP patients. No ratios were >3 : 1 or <1 : 3.

The kappa : lambda (*κ*/*λ*) ratio for B lymphocytes in IRP patients was detected by FCM. No samples produced a *κ*/*λ* ratio of more than 3 : 1 or less than 1 : 3 (Figure 2). CD21 and CD23 expression on CD19+ B lymphocyte surfaces was detected using FCM. CD21 expression levels in IRP patients with anti-EBVCA IgM positivity (mean, 88.58 ± 1.391%; *n* = 17) were higher (*P* = 0.005) than those in IRP patients with anti-EBVCA IgM negativity (mean, 79.84 ± 2.541%; *n* = 17). CD23 expression levels in IRP patients with anti-EBVCA IgM positivity (mean, 58.35 ± 4.705%; *n* = 18) were substantially higher (*P* = 0.008) than those in IRP patients with anti-EBVCA IgM negativity (mean, 41.35 ± 3.902%; *n* = 21).

**Figure 3:**
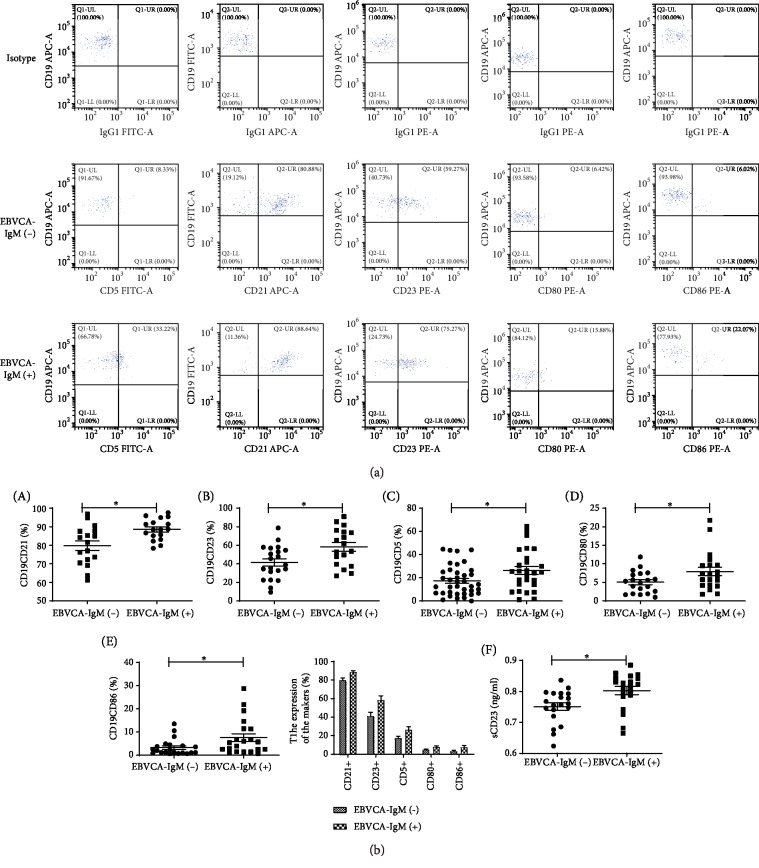
(a) Expression levels of activator antigens of CD19+ B lymphocytes. Expression of activator antigens of CD19+ B lymphocytes detected using flow cytometry. (b) Expression levels of (A) CD21, (B) CD23, (C) CD5, (D) CD80, and (E) CD86 on CD19+ B lymphocytes in IRP patients with anti-EBVCA IgM negativity and with anti-EBVCA IgM positivity. (F) Serum levels of soluble CD23 (sCD23) in IRP patients with anti-EBVCA IgM negativity and with anti-EBVCA IgM positivity.^∗^*P* < 0.05.

CD5, CD80, and CD86 expression on CD19+ B lymphocyte surfaces was also detected using FCM. CD5 expression levels were significantly higher in IRP patients with anti-EBVCA IgM positivity (mean, 26.47 ± 3.358%; *n* = 27) than in IRP patients with anti-EBVCA IgM negativity (mean, 17.41 ± 2.098%; *n* = 38; *P* = 0.0205). Similar trends were observed for CD80 and CD86 expression levels. The mean CD80 expression levels for IRP patients with anti-EBVCA IgM positivity and with anti-EBVCA IgM negativity were 7.948 ± 1.165% (*n* = 20) and 5.044 ± 0.6529% (*n* = 20; *P* = 0.036), respectively, and the mean CD86 expression levels were 7.611 ± 1.682% (*n* = 21) and 3.350 ± 0.7604% (*n* = 21; *P* = 0.0262), respectively. Serum levels of sCD23 were determined using ELISA. IRP patients with anti-EBVCA IgM positivity had significantly higher (*P* = 0.0062) serum levels of sCD23 (mean, 0.803 ± 0.013 ng/mL; *n* = 20) than IRP patients with anti-EBVCA IgM negativity (mean, 0.751 ± 0.012 ng/mL; *n* = 20; Figure 3).

**Figure 4:**
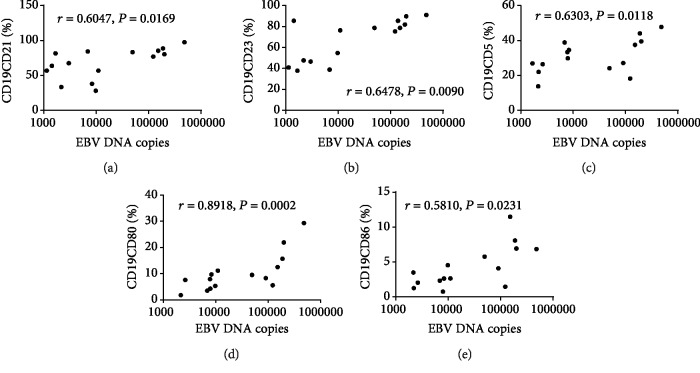
Correlation between EBV-DNA copy numbers and activator antigen abundances on B lymphocyte surfaces in IRP patients. Correlation of EBV-DNA copy numbers with (a) CD21, (b) CD23, (c) CD5, (d) CD80, and (e) CD86 abundances on CD19+ B lymphocyte surfaces in IRP patients. *n* = 15.

Next, we correlated CD21, CD23, CD5, CD80, and CD86 expression on CD19+ B lymphocyte surfaces in IRP patients with EBV-DNA copy numbers (*n* = 15). The results revealed that EBV-DNA copy numbers were positively correlated with CD21 (*r* = 0.6047, *P* = 0.0169), CD23 (*r* = 0.6478, *P* = 0.009), CD5 (*r* = 0.6303, *P* = 0.0118), CD80 (*r* = 0.8918, *P* = 0.0002), and CD86 (*r* = 0.5810, *P* = 0.0231) expression (Figure 4).

### 3.3. Relationship between EBV and Clinical Prognosis in IRP Patients

**Figure 5:**
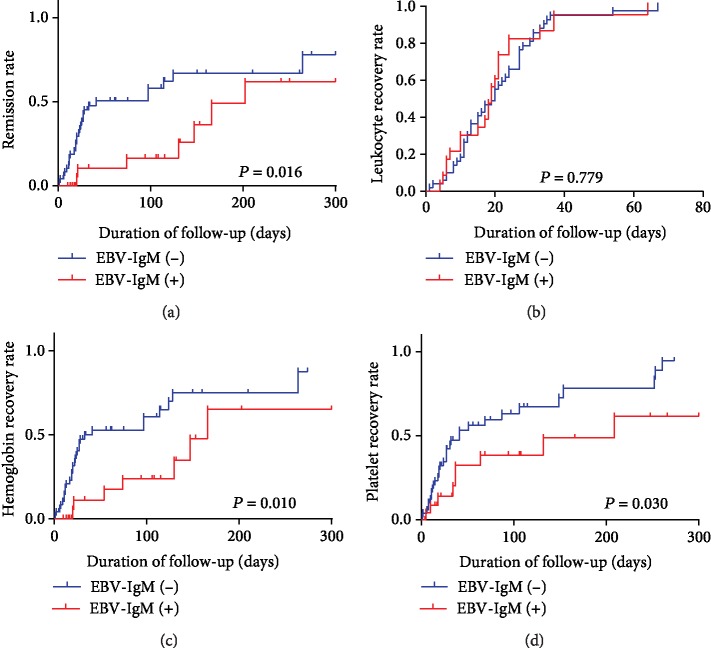
Effects of EBV infection on IRP patients. (a) The remission times, (b) leukocyte recovery times, (c) hemoglobin recovery times, and (d) platelet recovery times of IRP patients with anti-EBVCA IgM negativity and with anti-EBVCA IgM positivity.

IRP patients with anti-EBVCA IgM positivity took a significantly longer time to attain remission than IRP patients with anti-EBVCA IgM negativity. The remission curves also differed significantly (*P* = 0.016). However, leukocyte recovery times did not differ significantly between the two groups (*P* = 0.779). By contrast, hemoglobin (*P* = 0.010) and platelet (*P* = 0.030) recovery took a significantly longer time in IRP patients with anti-EBVCA IgM positivity than in IRP patients with anti-EBVCA IgM negativity (Figure 5).

## 4. Discussion

In recent years, cases exhibiting persistent hemocytopenia not attributable to hematological or nonhematological diseases have been described as idiopathic cytopenia of undetermined significance (ICUS) [11]. In our previous study, autoantibodies were found in some ICUS patients, and these antibodies may facilitate damage to hematopoietic cells in the bone marrow via phagocytosis by macrophages [12]. Dysregulation of Tfh cell function or expression of Tfh cell-associated molecules could contribute to the pathogenesis of IRP [4]. These ICUS patients responded well to corticosteroids and/or high-dose intravenous immunoglobulin. We thus termed this disorder “immunorelated hemocytopenia” (IRH), also known as BMMNC-Coombs test-positive hemocytopenia. Further, the production of autoantibodies in these patients may be caused by hyperfunction of B lymphocytes [5]. We had previously looked for IgM and IgG antibodies on the membranes of various bone marrow cells of ICUS patients using the BMMNC-Coombs test, FCM, and immunofluorescence analysis and determined that some patients had autoantibodies that led to immune dysfunction resulting in the destruction of hematopoietic cells in the bone marrow, causing IRH [13–15]. IRH is a type of hemocytopenia regarded as an autoimmune disease caused by unknown autoantibodies that may suppress hematopoietic cells in the bone marrow. Eventually, IRH leads to the clinical manifestation of different degrees of anemia, bleeding, and infection [16, 17]. IRP is a type of IRH. Its main manifestation is pancytopenia. IRP is thought to be caused by abnormalities in the numbers, subsets, and functions of CD19+ B lymphocytes. Additionally, abnormal activation of B lymphocytes leads to IRP pathogenesis [5]. However, the role of CD19+ B lymphocytes in disease progression is still unclear. Certain environmental factors, such as damage from chemicals, drugs, viruses, or antigens, may be involved in or initiate IRP pathogenesis by triggering lymphocyte activation and further immune responses in the body. In particular, viral infection is widely examined as a potential factor. In this study, we investigated whether viral infection caused the abnormal activation of B lymphocytes and thus, IRP pathogenesis.

EBV is associated with several autoimmune diseases such as SLE, RA, MS, autoimmune thyroiditis, inflammatory bowel diseases, insulin-dependent diabetes mellitus, Sjögren's syndrome, systemic sclerosis, myasthenia gravis, and autoimmune liver disease [8, 18–20]. Therefore, it is worthwhile to explore how EBV infection is established and maintained in IRP patients and how it activates B lymphocytes to facilitate IRP pathogenesis. Various methods can be used to detect EBV. Currently, EBVCA-IgG, EBVCA-IgM, and EBV-DNA are most commonly used to diagnose EBV infection in the laboratory [21, 22]. The primary antibodies for acute and past EBV infections are the IgM and IgG antibodies specific to the EBV capsid antigen, respectively. In patients with acute EBV infection, EBVCA-IgM titers peak at 3–4 weeks after infection, whereas EBVCA-IgG titers peak at 1–2 months after infection, then decrease slightly and remain in the body for the rest of a patient's life. Our results revealed that EBV infection is associated with IRP pathogenesis, as the positive rate of EBVCA-IgM and CD19+ B lymphocyte EBV-DNA copy in the IRP group were significantly higher than those in the control group (*P* < 0.05). CD19+ B lymphocyte EBV-DNA copies were also more abundant in IRP patients than in control subjects (*P* < 0.05).

Binding of the EBV membrane glycoprotein gp350/220 to CD21 on B lymphocytes likely initiates adsorption, capping, and endocytosis [23, 24]. These are accompanied by increased mRNA synthesis, blast transformation, homotypic cell adhesion, surface CD23 expression, and IL-6 production [25]. It has been demonstrated that EBV infection of target cells depends on the density of CD21 molecules [26]. EBV also induces sustained CD23 overexpression in B lymphocytes. CD23 is a multifunctional molecule that stimulates the growth of B lymphocytes [27]. The activation of CD23 molecules, and EBV binding to CD21, can promote the proliferation of B lymphocytes [7]. Many proteins can promote cell proliferation and activation [28]. Our results revealed that CD19+CD21+ and CD19+CD23+ B cell levels and sCD23 serum levels were significantly higher in patients with anti-EBVCA IgM positivity than in patients with anti-EBVCA IgM negativity. The possible mechanism of action is as follows: After B lymphocytes in IRP patients are infected with EBV, transactivator proteins EBNA2 and LMP1 are produced to activate the CD21 and CD23 genes, which results in sustained high expression of CD21 and CD23 in B cells. Once separated, CD23 becomes sCD23, the autocrine B cell growth factor, which binds to CD21 and activates the production of tyrosine protein kinase. This leads to the production and proliferation of antibody-releasing B lymphocytes, resulting in immune responses and damage. We also calculated the *κ*/*λ* ratios for CD19+ B lymphocytes in IRP patients. Ratios were not >3 : 1 or <1 : 3. Hence, the abnormal activation of B lymphocytes in IRP patients was polyclonal rather than clonal.

As previously stated, EBV infection of B lymphocytes leads to their activation. After the lymphocytes are activated, the expression of some antigens with related functions, which can receive extracellular signals, increases. CD80 and CD86 belong to the B7 family, members of which are the principal active antigens on B lymphocyte surfaces [29, 30]. In addition, CD5 of activated B lymphocytes was unregulated. CD5 lymphocytes are considered the principal cells that produce autoantibodies against IgM. They can form an authoritative cell bank that is linked to the development of autoimmune diseases [31–33]. Our results revealed that CD19+CD5+, CD19+CD80+, and CD19+CD86+ B cell abundances were significantly higher in patients with anti-EBVCA IgM positivity than in patients with anti-EBVCA IgM negativity. This may be because EBV activates B lymphocytes upon infection, leading to increases in the abundances of their activator antigens CD5, CD80, and CD86, which play major roles in maintaining immune stability. Abnormal expression of CD80 and CD86 may lead to the initiation and exacerbation of autoimmune diseases. CD80 and CD86 are mainly expressed in activated B lymphocytes. They supply the second signal for T cell activation and induce the T cells to increase CD5+ B lymphocyte abundance and autoantibody production. The body then produces autoantibodies against hematopoietic cells in the bone marrow, thereby inhibiting and/or destroying the hematopoietic cells, which leads to hematopoietic failure in the bone marrow and peripheral blood cell decline. These events may contribute to IRP pathogenesis.

After a year of follow-up with the IRP patients, we found that the remission rate of patients with anti-EBVCA IgM positivity was significantly lower than that of patients with anti-EBVCA IgM negativity, indicating that EBV infection delays IRP remission. This increases the patient's economic expenses and reduces the quality of life. However, white blood cell count did not differ significantly between the two groups. The granulocyte colony-stimulating factor may have caused the leukocyte number to increase in leukopenia patients, which would then affect our statistical analyses.

In summary, the positive rate of EBVCA-IgM and CD19+ B lymphocyte EBV-DNA copy in the IRP group were significantly higher than those in the control group. CD19+ B lymphocyte EBV-DNA copy numbers were significantly higher in IRP patients than in control subjects. Expression levels of CD21, CD23, CD5, CD80, and CD86 on CD19+ B cell surfaces were significantly higher in IRP patients with anti-EBVCA IgM positivity than in IRP patients with anti-EBVCA IgM negativity. EBV infection may be important for activating CD19+ B lymphocytes and may cause further disruption to bone marrow hematopoiesis in IRP patients. This article is a retrospective study. We summarized its characteristics from clinical manifestations and found regular guidelines to apply to clinical practice. However, we have not done animal experiments, verify the other functional changes of B lymphocytes after EB virus infection, and clarify the mechanism by which EB virus affects the abnormal changes of B lymphocytes, which is our next work plan and focus.

## Figures and Tables

**Table 1:** The positive rate of EBVCA-IgG and EBVCA-IgM in IRP patients and control subjects.

	*n*	EBVCA-IgG	EBVCA-IgM
IRP patients	72	71 (98.6%)	31 (43.1%)
Control subjects	36	35 (97.2%)	8 (22.2%)
*χ* ^2^		0.254	4.515
*P*		0.613	0.033

**Table 2:** The positive rate of CD19+ B lymphocyte EBV-DNA copies in IRP patients and control subjects.

	*n*	EB-DNA
IRP patients	51	23 (45.1%)
Control subjects	27	6 (22.2%)
*χ* ^2^		3.955
*P*		0.047

**Table 3:** The number of CD19+ B lymphocyte EBV-DNA copies in IRP patients and control subjects.

	*n*	EB-DNA load
Control subjects	6	7.277 × 10^3^ (2.557 × 10^3^-1.9775 × 10^4^)
Remission IRP patients	11	7.781 × 10^3^ (2.191 × 10^3^-4.1210 × 10^4^)
Newly diagnosed IRP patients	12	9.646 × 10^3^ (2.660 × 10^3^-1.5475 × 10^5^)^∗^^#^

Data was presented as the median. ^∗^Compared with remission IRP patients, *P* < 0.05. ^#^Compared with control subjects, *P* < 0.05.

## Data Availability

The data used to support the findings of this study are available from the corresponding author upon request.

## References

[1] Hao S., Fu R., Wang H., Shao Z. (2017). Screening novel autoantigens targeted by serum IgG autoantibodies in immunorelated pancytopenia by SEREX. *International journal of hematology*.

[2] Fu R., Liu H., Wang Y. (2014). Distinguishing immunorelated haemocytopenia from idiopathic cytopenia of undetermined significance (ICUS): a bone marrow abnormality mediated by autoantibodies. *Clinical and experimental immunology*.

[3] Shao Y., Fu R., Liu H. (2015). IgG autoantibody subclasses altered in immuno-related hemocytopenia. *Cellular immunology*.

[4] Yu H., Zhang J., Fu R. (2013). Increased frequency of bone marrow T follicular helper cells in patients with immune-related pancytopenia. *Clinical & developmental immunology*.

[5] Fu R., Shao Z., He H. (2002). Quantity and apoptosis-related protein level of B lymphocyte in patients with immunorelated pancytopenia. *Zhonghua xue ye xue za zhi = Zhonghua xueyexue zazhi*.

[6] Epstein M. A., Achong B. G., Barr Y. M. (1964). VIRUS PARTICLES IN CULTURED LYMPHOBLASTS FROM BURKITT'S LYMPHOMA. *The Lancet*.

[7] Rowe D. T. (1999). Epstein-Barr virus immortalization and latency. *Frontiers in bioscience: a journal and virtual library*.

[8] Lossius A., Johansen J. N., Torkildsen Ø., Vartdal F., Holmøy T. (2012). Epstein-Barr Virus in Systemic Lupus Erythematosus, Rheumatoid Arthritis and Multiple Sclerosis—Association and Causation. *Viruses*.

[9] Taylor G. S., Long H. M., Brooks J. M., Rickinson A. B., Hislop A. D. (2015). The immunology of Epstein-Barr virus-induced disease. *Annual review of immunology*.

[10] Cui J., Yan W., Xu S. (2018). Anti-Epstein-Barr virus antibodies in Beijing during 2013-2017: what we have found in the different patients. *Plos One*.

[11] Wimazal F., Fonatsch C., Thalhammer R. (2007). Idiopathic cytopenia of undetermined significance (ICUS) versus low risk MDS: the diagnostic interface. *Leukemia research*.

[12] Sun L. F., Han B., Wu Q. Q. (2013). Immune mechanism and clinical significance of macrophage to medullary hematopoietic injury of immune-related hematocytopenia patients. *Chinese medical journal*.

[13] Wang Y. H., Fu R., Shao Z. H. (2014). A pilot study of memory B lymphocytes in relapsed immune-related pancytopenia patients. *Clinical laboratory*.

[14] Shao Y., Qi X., Fu R. (2018). Demonstration of IgG subclass (IgG1 and IgG3) in immuno-related hemocytopenia. *Clinical laboratory*.

[15] Liu H., Fu R., Wang Y. (2013). Detection and analysis of autoantigens targeted by autoantibodies in immunorelated pancytopenia. *Clinical & developmental immunology*.

[16] Wang Y. H., Fu R., Dong S. W., Liu H., Shao Z. H. (2014). Erythroblastic islands in the bone marrow of patients with immune-related pancytopenia. *PloS one*.

[17] Li Y., Wang Y., Liu H. (2018). Lower level of IL-35 and its reduced inhibition in Th17 cells in patients with bone marrow mononuclear cells Coombs test-positive hemocytopenia. *Molecular medicine reports*.

[18] Rasmussen N. S., Draborg A. H., Nielsen C. T., Jacobsen S., Houen G. (2015). Antibodies to early EBV, CMV, and HHV6 antigens in systemic lupus erythematosus patients. *Scandinavian journal of rheumatology*.

[19] Westergaard M. W., Draborg A. H., Troelsen L., Jacobsen S., Houen G. (2015). Isotypes of Epstein-Barr virus antibodies in rheumatoid arthritis: association with rheumatoid factors and citrulline-dependent antibodies. *BioMed Research International*.

[20] Dittfeld A., Gwizdek K., Michalski M., Wojnicz R. (2016). A possible link between the Epstein-Barr virus infection and autoimmune thyroid disorders. *Central-European Journal of Immunology*.

[21] Leung E., Shenton B. K., Jackson G., Gould F. K., Yap C., Talbot D. (2002). Use of real-time PCR to measure Epstein-Barr virus genomes in whole blood. *Journal of immunological methods*.

[22] Ruiz G., Peña P., de Ory F., Echevarría J. E. (2005). Comparison of commercial real-time PCR assays for quantification of Epstein-Barr virus DNA. *Journal of clinical microbiology*.

[23] Nemerow G. R., Wolfert R., McNaughton M. E., Cooper N. R. (1985). Identification and characterization of the Epstein-Barr virus receptor on human B lymphocytes and its relationship to the C3d complement receptor (CR2). *Journal of virology*.

[24] Tanner J., Weis J., Fearon D., Whang Y., Kieff E. (1987). Epstein-Barr virus gp350/220 binding to the B lymphocyte C3d receptor mediates adsorption, capping, and endocytosis. *Cell*.

[25] Gordon J., Walker L., Guy G., Brown G., Rowe M., Rickinson A. (1986). Control of human B-lymphocyte replication. II. Transforming Epstein-Barr virus exploits three distinct viral signals to undermine three separate control points in B-cell growth. *Immunology*.

[26] Haan K. M., Aiyar A., Longnecker R. (2001). Establishment of latent Epstein-Barr virus infection and stable episomal maintenance in murine B-cell lines. *Journal of virology*.

[27] Luo H. Y., Hofstetter H., Banchereau J., Delespesse G. (1991). Cross-linking of CD23 antigen by its natural ligand (IgE) or by anti-CD23 antibody prevents B lymphocyte proliferation and differentiation. *The Journal of immunology: official journal of the American Association of Immunologists*.

[28] Wang F., Li Y., Shan F. (2019). Upregulation of JMJD2A promotes migration and invasion in bladder cancer through regulation of SLUG. *Oncology reports*.

[29] Peggs K. S., Allison J. P. (2005). Co-stimulatory pathways in lymphocyte regulation: the immunoglobulin superfamily. *British journal of haematology*.

[30] Bhatia S., Edidin M., Almo S. C., Nathenson S. G. (2006). B7-1 and B7-2: similar costimulatory ligands with different biochemical, oligomeric and signaling properties. *Immunology letters*.

[31] Lee K. W., Lee S. H., Kim H. J., Kim J. M., Choi Y. M., Motomura M. (1999). Experimental autoimmune myasthenia gravis and CD5+ B-lymphocyte expression. *Journal of Korean medical science*.

[32] Cantaert T., Doorenspleet M. E., Francosalinas G. (2012). Increased numbers of CD5+ B lymphocytes with a regulatory phenotype in spondylarthritis. *Arthritis and rheumatism*.

[33] Van der Weerd K., Van Hagen P. M., Schrijver B. (2013). The peripheral blood compartment in patient with Graves’ disease: activated T lymphocytes and increased transitional and pre-naive mature B lymphocytes. *Clinical and experimental immunology*.

